# Linking evidence for targeted blood biomarkers in post-stroke cognitive impairment and dementia

**DOI:** 10.3389/fstro.2024.1491542

**Published:** 2025-01-07

**Authors:** Jinny Hong, Katherine Mun, Kyle C. Kern, Marissa Thirion, Jason D. Hinman

**Affiliations:** ^1^Chicago College of Osteopathic Medicine, Midwestern University, Downers Grove, IL, United States; ^2^Department of Neurology, West Los Angeles Veteran Affairs Medical Center, Los Angeles, CA, United States; ^3^Department of Neurology, David Geffen School of Medicine, University of California, Los Angeles, Los Angeles, CA, United States

**Keywords:** post-stroke, cognitive impairment, dementia, vascular dementia, biomarkers, neuroinflammation, neuroaxonal injury, vascular injury

## Abstract

With improvements in acute stroke treatment and more patients surving the acute stroke period, the identification and prognostication of post-stroke disability is paramount. Post-stroke cognitive impairment and dementia (PSCID) severely impacts the morbidity and mortality of stroke survivors. While clinical factors and imaging are useful in identifying patients at risk for PSCID, blood-based biomarkers are sorely needed to provide cost-effective identification and prognostication for patients at greatest risk. Furthermore, blood-based biomarkers can inform the biologic basis for PSCID and lead to potential treatment targets. This narrative review attempts to summarize currently available research on the use of fluid biomarkers to measure and quantify PSCID using a framework proposed for use in the DISCOVERY Network study of PSCID. In this framework, blood biomarkers are divided into broad pathologic categories including inflammation, neurodegeneration, neuroaxonal injury, and vascular injury. Key biomarkers that have been proposed as relevant to PSCID include interleukin-6, C-reactive protein, β-amyloid 42:40 ratio, neurofilament light chain, and 10 angiogenic molecules. Critical to the assessment of prior studies includes defining the sample collection period and cognitive assessment period of prior studies to assess the temporal pattern of biomarker levels in relation to an incident stroke event. In addition to this comprehensive review, we performed a protein-protein network analysis of the putative blood biomarkers for PSCID and (surprisingly) find they exist in a highly connected protein-protein interaction network centered on inflammatory and neurodegenerative biomarkers suggesting shared biology underlies the pathogenesis of PSCID. Both the literature and this network analysis point to a role for the use of combinatorial blood biomarkers as a methodology to enhance the specificity and sensitivity of putative prognostic biomarkers for PSCID. This review highlights the emerging role for blood biomarkers in evaluating risk for PSCID while also informing the underlying biology that creates synergy between stroke and dementia.

## 1 Introduction

**Figure 1:**
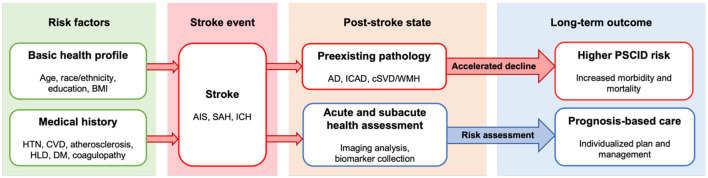
Overview of stroke progression and post-stroke outcome. Health profile and medical history contribute to pathogenesis of stroke and may elevate risk for post-stroke cognitive impairment and dementia. Post-stroke patient outcome may be influenced by presence of preexisting pathology, which elevates risk for PSCID. Acute to subacute period health status assessment guided by imaging and biomarkers can facilitate risk assessment, early intervention, and management as indicated by the blue arrow. AD, Alzheimer's disease; AIS, acute ischemic stroke; BMI, body mass index; cSVD, cerebral small vessel disease; CVD, cardiovascular disease; DM, diabetes mellitus; HLD, hyperlipidemia; HTN, hypertension; ICAD, intracranial atherosclerotic disease; ICH, intracranial hemorrhage; PSCID, poststroke cognitive impairment and dementia; SAH, subarachnoid hemorrhage; WMH, white matter hyperintensity.

Stroke is the second leading cause of death and disability worldwide due to its high mortality rate and severe neurologic complications (Tsao et al., 2023). Stroke arises from vascular injuries such as infarction or hemorrhage within the central nervous system, often resulting in disability and diminished quality of life (Chen et al., 2023; Cucchiara et al., 2019). Of the many complications of stroke, post-stroke cognitive impairment and dementia (PSCID) is the most common. PSCID describes post-stroke cognitive dysfunction or dementia-like decline that persists for a minimum of 1 year from the onset of stroke (Rost et al., 2021, 2022), and it affects 15–70% of stroke patients depending on the time of assessment, stroke type, infarct size and location, patient health profile, and other various factors (Rost et al., 2022; Douiri et al., 2013; Pendlebury and Rothwell, 2019; Lo et al., 2019; Wong et al., 2012; Filler et al., 2024). PSCID poses a particular challenge because of its high prevalence, complex pathogenesis, and poor patient outcomes (Kjörk et al., 2016; Droś et al., 2024) that can be influenced by multiple confounding factors (Figure 1).

It is postulated that the onset and progression of PSCID may relate to vascular injury, inflammation, neuroaxonal injury, neurodegeneration, or any combination of these pathologies, but the precise mechanism largely remains unclear. The problem is further complicated by PSCID's multiple etiologies and contributors. Acute ischemic stroke (AIS), intracranial hemorrhage (ICH), and subarachnoid hemorrhage (SAH) can all result in PSCID, but the difference in severity and pathophysiology of PSCID behind each stroke type is poorly understood. Strokes often occur in patients with co-morbidities that also affect brain health, including Alzheimer's disease (AD), intracranial atherosclerotic disease (ICAD), cerebral small vessel disease (cSVD)/white matter hyperintensities (WMH), and heart disease. The effect of these pre-existing pathologies on the progression of PSCID has been understudied as well (Rost et al., 2022). Due to these gaps in knowledge, several large scale studies are focused on elucidating potential mechanisms or testing interventions for PSCID including the Oxford Vascular study (OXVASC), the DISCOVERY Network study (Determinants of Incident Stroke Cognitive Outcomes and Vascular Effects on Recovery), and the LACI-2 Trial (Lacunar Intervention Trial-2), among others reviewed elsewhere (El Husseini et al., 2023). In this review, we focus on the blood-based biomarkers proposed in DISCOVERY and provide evidence for a connected protein network that may aid our overall understanding of the biologic pathways underlying PSCID.

Investigations of blood-based biomarkers are positioned to play a central role in characterizing PSCID with several advantages that are not present in other types of biomarkers such as cerebrospinal fluid (CSF) and imaging biomarkers. While CSF biomarkers hold significant diagnostic value for intracranial pathologies, collection via lumbar puncture is invasive with risk for complications including back pain, headache, intracranial hypotension, and even epidural hematoma (Reis et al., 2024; Bodilsen et al., 2020; Lyons et al., 2022), particularly for stroke patients who require antithrombotics for secondary prevention. Similarly, imaging biomarkers, including magnetic resonance imaging (MRI) or positron emission tomography (PET), are limited by cost, availability, and contraindications such as pacemakers and metallic foreign bodies (Márquez and Yassa, 2019; Hansson, 2021; Ghadimi and Sapra, 2023). In contrast, venipuncture is widely available and blood biomarkers are both cost effective and minimally invasive, making them the ideal screening tool. They also convey critical biologic information about the pathogenesis of PSCID. Guided care and management specific to each patient's biomarker pattern may lead to better outcomes for those identified as higher risk for PSCID (Hinman et al., 2017; Merriman et al., 2018; Demurtas et al., 2020). In this review, we will characterize existing evidence for several major classifications of biomarkers belonging to the categories of vascular injury, inflammation, neuroaxonal injury, and neurodegeneration as per the DISCOVERY study protocol (Rost et al., 2022).

## 2 Relevant biomarkers of vascular injury

Chronic cerebrovascular injuries, such as cSVD, increase the risk of stroke and dementia through multiple pathologic processes including hypertension-induced hyalinosis and vascular sclerosis, hypoperfusion, alterations in vascular wall permeability, and inflammation (Chojdak-Łukasiewicz et al., 2021; Biesbroek and Biessels, 2023). When vascular wall injury triggers intravascular coagulation and angiogenesis, biomarkers of endothelial dysfunction are upregulated. Some of the well-known CSF and plasma markers are cell adhesion molecules, vascular endothelial growth factor (VEGF) isoforms, and placental growth factor (PlGF) (Jaime Garcia et al., 2023; Winder et al., 2020; Hinman et al., 2023; Wang Y. et al., 2021; Hansson et al., 2019). Markers reflective of the coagulation cascade include von Willebrand factor (vWF) and fibrinogen (Jaime Garcia et al., 2023).

VEGF refers to a protein family that stimulates angiogenesis and neuroplasticity (Storkebaum et al., 2004; Moon et al., 2021) and is known for its pathogenic involvement in cancer (Shaw et al., 2024). VEGF is upregulated following a vascular occlusion. The first rise occurs 2–6 h after stroke and is followed by a second rise occuring during day 3–7 (Moon et al., 2021). While VEGF-A is the predominantly studied isoform, other relevant isoforms include VEGF-B, VEGF-C, and VEGF-D. Release of VEGF-A by endothelial and other cell types stimulates angiogenesis of nearby blood vessels leading to increased blood-brain barrier permeability to augment the delivery of oxygen and nutrients to injured tissue, although high levels are associated with adverse outcomes as well-associated with the increased BBB leakage (Hu et al., 2022). Due to its involvement in angiogenesis and neuroplasticity, VEGF-A have been studied as a marker for cSVD, AD (Yu et al., 2016; Hohman et al., 2015), and ischemic stroke (Hu et al., 2024; Seidkhani-Nahal et al., 2021) with emphasis on its prognostic value. Bhasin et al.'s (2019) study demonstrates that patients with AIS and elevated serum VEGF-A acutely were more susceptible to post-stroke disability, including functional status at 3 months. Escudero et al. arrived at a similar conclusion regarding the prognostic value of VEGF-A in acute ischemic stroke. Higher serum VEGF-A at 24–48 h from stroke onset increased the risk for moderate to severe disability or death at 6 months post-stroke. Prognostic performance of VEGF-A improved further when combined with IL-6 and CRP levels (Escudero et al., 2020). In a study by Zhang et al. (2020), elevated serum VEGF-A in acute ischemic stroke was associated with an unfavorable 90-day prognosis. Włodarczyk et al. (2023) found that lower plasma VEGF-A was associated with improvement in depressive symptoms 3 weeks after stroke.

Based on currently available studies, VEGF-A shows strong potential as a biomarker for PSCID, while other isoforms such as VEGF-C and D still warrant further investigation. In Prodjohardjono et al.'s (2020) study, the authors found that at 5 days post-ischemic stroke serum VEGF-A increased above 519.8 pg/mL, in combination with larger infarct volume, was associated with a 5-fold higher risk of cognitive impairment at 3 months. In a study by Åberg et al., however, findings were mixed. In their study, serum VEGF (isoform not specified) at median of 4 days post-ischemic stroke was non-significantly elevated (median of 573.5 pg/mL) in patients with 3-month functional outcome; however, higher VEGF (median of 5,553.7 pg/mL) at 3 months correlated with worse functional outcome 2 years after stroke (Åberg et al., 2020). How VEGF-A relates to ischemic stroke subtype may be more nuanced. Matsuo et al. (2013) found that higher plasma VEGF-A (median of 681.0 pg/mL) was associated with poor functional outcome at day 90 after cardioembolic stroke, but they found the opposite relationship with atherothrombotic stroke (mean of 619.0 pg/mL) with non-significant findings in lacunar infarction and other ischemic stroke subtypes. Summarized results are available in Table 1. Findings suggest that VEGF-A may be a predictor of overall patient outcomes, especially in the case of cardioembolic ischemic stroke, but results are inconsistent when it comes to association with PSCID and functional outcome, likely due to VEGF-A's pleiotropic role in human body. Further investigation of other VEGF isoforms such as VEGF-C and VEGF-D may reveal better specificity for PSCID. Between the two, VEGF-D is most promising due to its involvement in neuronal stability and memory consolidation (Stacker and Achen, 2018; Hemstedt et al., 2017).

**Table 1:** Abbreviated study results of fluid biomarkers.

**Category**	**Biomarker**	**Fluid type**	**Country**	**Collection period**	**Cognitive assessment period**	**Correlation with cognitive impairment**
Vascular Injury	VEGF-A	Serum	China, *N* = 225 (Zhang et al., 2020)	Admission	Mont	Positive
			India, *N* = 250 (Bhasin et al., 2019)	Day 0–7	Month 3	
			Chile, *N* = 45 (Escudero et al., 2020)	Day 1–2	Month 6	
			Indonesia, *N* = 56 (Prodjohardjono et al., 2020)	Day 5	Month 3	
			Sweden, *N* = 492 (Åberg et al., 2020)	Month 3	Year 2	
			Sweden, *N* = 492 (Åberg et al., 2020)	Day 0–7	Month 3	Non-significant
		Plasma	Japan, *N* = 171 (Matsuo et al., 2013)	Day 0–14	Month 3	Mixed
	Fibrinogen	Plasma	China, *N* = 134 (Liu et al., 2019)	Day 0–7	Month	Positive
			Sweden, *N* = 268 (Pedersen et al., 2018)	Month 3	Year 7	
			Italy, *N* = 128 (Di Napoli et al., 2001)	Day 1	Year 1	Non-significant
Inflammation	IL-1β	Serum	Singapore, *N* = 243 (Narasimhalu et al., 2015)	Month 1–2	Year 3-5	Non-significant
		Plasma	Norway, *N* = 455 (Sandvig et al., 2023)	Month 18	Month 36	Positive
	IL-1Ra	Plasma	Norway, *N* = 455 (Sandvig et al., 2023)	Month 3	Month 18, 36	Positive
	IL-6	Serum	Lithuania, *N* = 78 (Bunevicius et al., 2015)	Admission	Discharge	Positive
			Russia, *N* = 92 (Kulesh et al., 2018)	Day 4–21	Day 7–14	
			Singapore, *N* = 243 (Narasimhalu et al., 2015)	Month 1–2	Month 1–2	Non-significant
		Plasma	China, *N* = 1,003 (Wang et al., 2022)	Admission	Year	Positive
			Norway, *N* = 455 (Sandvig et al., 2023)	Day 3–6	Month 3, 18, 36	
			Norway, *N* = 455 (Sandvig et al., 2023)	Month 18	Month 36	
	IL-7	Plasma	Norway, *N* = 455 (Sandvig et al., 2023)	Day 3–6	Month 36	Positive
	IL-8	Serum	Singapore, *N* = 243 (Narasimhalu et al., 2015)	Month 1–2	Month 1–2	Positive
		Plasma	Norway, *N* = 455 (Sandvig et al., 2023)	Day 3–6	Month 36	Positive
	IL-12	Serum	Singapore, *N* = 243 (Narasimhalu et al., 2015)	Month 1–2	Year 3–5	Positive
	CRP	Serum	Israel, *N* = 368 (Kliper et al., 2013)	Day 0–3	Year	Positive
			Italy, *N* = 128 (Di Napoli et al., 2001)	Day 1	Year 1	
			Singapore, *N* = 243 (Narasimhalu et al., 2015)	Month 1–2	Year 3–5	Non-significant
	hsCRP	Serum	Lithuania, *N* = 78 (Bunevicius et al., 2015)	Admission	Discharge	Positive
			Bulgaria, *N* = 47 (Weaver et al., 2002)	Day 0–2	Year 1	
			China, *N* = 134 (Liu et al., 2019)	Day 0–7	Month	Non-significant
			Sweden, *N* = 268 (Pedersen et al., 2018)	Month 3	Year 7	
	MMP-9	Serum	Egypt, *N* = 30 (Abdelnaseer et al., 2017)	Day 0–1	Day 3	Positive
			China, *N* = 180 (Pu et al., 2022)	Day 0–1	Month 3	
			Poland, *N* = 32 (Włodarczyk et al., 2023)	Week 5	Week 5	
			Egypt, *N* = 30 (Abdelnaseer et al., 2017)	Day 30	Day 30	Negative
Neuroaxonal Injury	NfL	Serum	Germany, *N* = 211 (Uphaus et al., 2019)	Admission	Day 90	Positive
			Germany, *N* = 30 (Tiedt et al., 2018)	Day 7	Month 6	
			Sweden, *N* = 595 (Pedersen et al., 2019)	Month 3	Year 7	
		Plasma	China, *N* = 1,694 (Wang Z. et al., 2021)	Day 0–2	Day 90	Positive
			USA, *N* = 314 (Gendron et al., 2020)	Day 1–3	Month 3–6	
Neuro-degeneration	Aβ42:Aβ40	Plasma	China, *N* = 55 (Chi et al., 2019)	Month 3	Year 1	Negative
			Taiwan, *N* = 136 (Huang et al., 2022)	Day 1–7	Month 3–12	Non-significant
	T-tau	Plasma	China, *N* = 55 (Chi et al., 2019)	Month 3	Year 1	Negative
			Taiwan, *N* = 136 (Huang et al., 2022)	Day 1–7	Month 3–12	Non-significant
	p-tau-181	Plasma	Taiwan, *N* = 136 (Huang et al., 2022)	Day 1–7	Month 3–12	Negative

Only the study results discussing serum or plasma biomarker levels were included in this table. Baseline level refers to level of biomarker upon admission or maximum of 48 h within the onset of stroke. All time points are described relative to stroke onset. Aβ42:Aβ40, ratio of β-amyloid 42:40; CRP, C-reactive protein; hsCRP, high-sensitivity C-reactive protein; IL-1β, interleukin-1 beta; IL-1Ra, interleukin-1 receptor antagonist; IL-6, interleukin-6; IL-7, interleukin-7; IL-8, interleukin-8; IL-12, interleukin-12; MMP9, matrix metalloproteinase-9; NfL, neurofilament light chain; p-tau-181, phosphorylated-tau-181; T-tau, total tau; EGF-A, vascular endothelial growth factor A.

PlGF is a pro-angiogenic molecule released at high levels during pregnancy but is also critical for cerebral angiogenesis during development, demonstrating synergism with VEGF (Carmeliet et al., 2001). Furthermore, in pre-clinical studies elevated PlGF promotes neoangiogenesis and neuroprotection after experimental ischemia (Autiero et al., 2003; Du et al., 2010; Liu et al., 2006). Plasma levels of PlGF are associated with vascular cognitive impairment and vascular dementia in a non-stroke population that is enriched for cSVD (Hinman et al., 2023). While angiogenesis is likely robust after ischemic stroke, the role of PlGF in predicting PSCID warrants further testing. Given its diagnostic value in measuring cSVD states and its known functions in regulating vascular permeability and cerebral angiogenesis, PlGF is also a strong potential candidate biomarker for assessing PSCID risk.

Plasma fibrinogen is a thrombotic enzyme involved in the coagulation pathway that activates fibrin assembly to promote clotting and wound healing. While fibrinogen plays a key role in our body to maintain health, its level is also positively associated with likelihood of venous thrombosis and cardiovascular disease including ischemic stroke as well-through mechanisms yet unclear (Wolberg, 2023). As an acute phase reactant whose level rises with inflammation, an elevated level of fibrinogen, along with CRP (Marioni et al., 2009), has been linked to faster cognitive decline in older adults as well (Rafnsson et al., 2007; Gallacher et al., 2010; Xu et al., 2007). Because elevated fibrinogen is a risk factor for increased stroke incidence, increased stroke severity, and poor stroke outcomes (Rothwell et al., 2004; Di Napoli and Singh, 2009), others have proposed that fibrinogen may be a useful biomarker for stroke and its complications. In a study by Liu et al. (2019), ischemic stroke patients with elevated plasma fibrinogen within a week of onset (median of 3.3 g/L) were more likely to experience cognitive impairment 3 months post-stroke. Similar prognostic value was found in a study by Pedersen et al. They found that for young patients (< 50 years) with stroke, higher plasma fibrinogen levels (median of 3.2 g/L) measured at ~3 months (median of 101 d), along with vWF and tPA, were associated with worse cognitive performance 7 years later (Pedersen et al., 2018). In contrast, Di Napoli et al. (2001) concluded increased plasma fibrinogen levels 24 h after ischemic stroke (mean of 4.76 g/L) to be non-significantly correlated with 1-year risks for death or subsequent vascular event; although patients in the highest tertile of fibrinogen levels (>6.17 g/L) had an increased relative risk by 4.18, the authors concluded that CRP was a better predictor after using regression analysis to calculate the independent association. The consistency of fibrinogen's positive association with a poor outcome suggests that fibrinogen is fit as a biomarker for PSCID although the overlap of median values in the above studies with the normal range (2.0–4.0) may make it a poor candidate for clinical use. Using a second biomarker such as CRP, both of which are acute phase reactants, with variable weights may be ideal to improve clinical prognostication.

## 3 Relevant biomarkers of inflammation

Broadly, neuroinflammation is recognized as one of the key players in the pathogenesis of neurodegenerative diseases such as AD (Twarowski and Herbet, 2023), Parkinson's disease (PD) (Morris et al., 2024), and multiple sclerosis (MS) (Rodríguez Murúa et al., 2022). There is also emerging evidence that chronic sterile inflammation associated with vascular risk factors may increase the risk for cerebrovascular injury and vascular cognitive impairment (Altendahl et al., 2020; Xiao et al., 2022; Sofia and Felipe, 2023). After acute cerebrovascular injury, a robust neuroinflammatory response is initiated by pro-inflammatory cytokines and other secreted factors, which drive further secondary inflammatory processes (Mun and Hinman, 2022). When this neuroinflammation becomes exaggerated and uncontrolled, recovery mechanisms can be disturbed, resulting in cognitive decline due to inflammation-driven secondary tissue injury (Mun and Hinman, 2022; Thapa et al., 2023; Stuckey et al., 2021). Based on this hypothesis, known neuroinflammatory biomarkers are reasonable candidates for quantifying the degree of neuroinflammation and secondary damage in patients after stroke. One established biomarker for neuroinflammatory cognitive decline is plasma C-reactive protein (CRP), which is associated with an increased risk of AD (Song et al., 2015; Brosseron et al., 2018), cSVD (Hilal et al., 2018), and PD (Umemura et al., 2015). Other candidates include pro- and anti-inflammatory serum interleukins, which are also associated with risk for AD (Sokolova et al., 2009; Khan et al., 2023), and CSF monocyte chemoattractant protein-1 (MCP-1), which is associated with risk for both AD (Sokolova et al., 2009) and PD (Santaella et al., 2020). Others include leukocytes counts or ratios, matrix metalloproteinase 9 (MMP-9), plasma myeloperoxidase (MPO) (Bawa et al., 2020), and soluble receptor for advanced glycation end products (RAGE) (Emanuele et al., 2005).

Interleukins (ILs) are a group of signaling cytokines released during immune response to modulate inflammation with various pro- or anti-inflammatory effects (Al-Qahtani et al., 2024). Emerging evidence has shown that pro-inflammatory cytokines, such as interleukin (IL)-6 and IL-8, are particularly promising as serum biomarkers for PSCID, as their levels show a positive trend among those experiencing cognitive decline (Rafnsson et al., 2007; Rani et al., 2022; Weaver et al., 2002). In the case of ischemic stroke patients, pro-inflammatory cytokines, IL-6 and tumor necrosis factor (TNF) in particular, are released within the first 24 h, with their levels positively associated with stroke severity and a poor prognosis; this process is followed by the release of anti-inflammatory cytokines to prevent secondary inflammatory degradation (Thapa et al., 2023). In the Nor-COAST study (Norwegian Cognitive Impairment After Stroke), levels of serum ILs, terminal C5b-9 complement complex (TCC), TNF, monocyte chemoattractant protein (MCP-1), and macrophage inflammatory protein (MIP) were measured at baseline, 3, and 18 months after ischemic stroke (Sandvig et al., 2023; Thingstad et al., 2018). Cognitive function was assessed at 3, 18, and 36 months after stroke using the Montreal Cognitive Assessment (MoCA). Upon analysis with mixed linear regression methods, elevated IL-6, TCC, and MIP-1α at baseline (median of 4 d) were associated with lower MoCA scores by 5–7 points at 3, 18, and 36 months after stroke. High baseline IL-1Ra was significantly associated with lower MoCA scores at 18 and 36 months only, and baseline IL-7, IL-8, and TNF were significantly associated with lower MoCA scores only at 36 months. Even though a non-significant negative relationship between the biomarker and the MoCA score could be observed for IL-1Ra, IL-7, IL-8, and TNF, the association didn't become significant until the later period at 18 or 36 months, likely due to the continued decline of the MoCA score. For biomarkers collected at 18 months, elevated IL-1β, IL-6, and MIP-1α were associated with lower MoCA scores (Sandvig et al., 2023). Authors concluded that IL-6 and MIP-1α measured at baseline and 18 months post-stroke were the most promising for assessing and monitoring post-stroke cognitive impairment. Studies conducted by Kulesh et al. and Wang et al. also support the utility of IL-1β (Kulesh et al., 2018) and IL-6 (Wang et al., 2022), respectively. However, conflicting results indicate that further investigation may be needed to support certain ILs. For example, in a 5-year follow-up study conducted at Singapore General Hospital, Narasimhalu et al. concluded that while high serum IL-8 at 1–2 months post-ischemic stroke (median of 47 d) was associated with baseline cognitive impairment, high serum IL-12 was associated with subsequent cognitive decline after 5 years; neither IL-1β nor IL-6 were found to be significantly associated (Narasimhalu et al., 2015). Using a backward stepwise elimination linear regression model, Rothenburg et al. reported an elevation in serum IL-6 within the first month after an ischemic stroke in patients who later developed poor cognitive function, but the result was not significant (Rothenburg et al., 2010). A likely explanation for mixed findings is the collection period of the cytokines. Pro-inflammatory cytokine levels rise rapidly soon after stroke, and they are heavily influenced by other pro-inflammatory processes within the body as well. It is likely that the specificity of pro-inflammatory cytokines to AIS decreases as time pass. Another explanation is that IL levels vary by numerous factors, including race, gender, activity level, and BMI. For example, baseline IL-6 is likely to be higher in minorities, women, sedentary individuals, and people with low extraversion resulting in decreased activity compared to the general population (Chapman et al., 2009; Qi et al., 2007; Amaral et al., 2015). Because baseline IL-6 may change depending on these factors, values are likely to have high standard deviation, which may make it difficult to appreciate the differences in IL-6 levels of two distinct groups if the study population is small. Despite the inconsistent findings, trends of pro-inflammatory cytokines such as IL-1β, IL-6, and IL-8 show positive association with likelihood for PSCID, which makes them worthwile targets for an investigation. Ideally, future studies should aim for an early fluid collection period and a large sample size to overcome the population variance in cytokine levels.

CRP is a pro-inflammatory, hepatic acute phase reactant protein. CRP is upregulated primarily by IL-6 and can cause secondary inflammatory damages through similar mechanisms (Plebani, 2023). Thus, like IL-6, elevated high-sensitivity CRP (hsCRP) has been associated with cognitive decline and recurrent stroke (Zheng and Xie, 2018; Elkind et al., 2014). Due to the agonist effect by IL-6 and their functional similarity, CRP shows promise as a biomarker for inflammation-mediated neurodegeneration. Among stroke patients, elevated CRP was associated with worse cognitive outcomes within 1 month after ischemic stroke as reported by Rothenburg et al. (2010). Bunevicius et al. (2015) also concluded that elevated serum hsCRP upon admission could predict worse cognitive function upon discharge of ischemic and hemorrhagic stroke patients. However, studies were mixed for longer-term prognostication of months to years after stroke. However, studies were mixed for longer-term prognostication of months to years after stroke. In a study conducted in Bulgaria, Alexandrova et al. concluded that patients with elevated serum hsCRP 48 h after an ischemic stroke were more likely to experience cognitive decline a year after the stroke (Alexandrova and Danovska, 2016). Patients in a mild-to-moderate cognitive deficit group also had significantly elevated hsCRP with median of 12 (4.3–35) mg/L compared to the group who retained normal cognitive function 1 year after stroke, whose median was 1.9 (1.2–2.5) mg/L. However, Narasimhalu et al. failed to show any association between CRP and PSCID (Narasimhalu et al., 2015). Mean serum CRP 1–2 months after the ischemic stroke was 9.77 mg/L with standard deviation of 20.41 mg/L in the moderate cognitive impairment without dementia group and 14.37 ± 23.92 mg/L in the dementia group, compared to 6.56 ± 12.41 mg/L in the normal group. As part of the TABASCO study (Tel Aviv Brain Acute Stroke Cohort), Kliper et al. observed that elevated serum CRP 72 h within an ischemic stroke was associated with cognitive impairment within 7 days of stroke, but the authors concluded that the association vanished upon adjustment for covariates (Assayag et al., 2012; Kliper et al., 2013). In a study conducted by Pedersen et al., hsCRP was investigated along with fibrinogen and tissue plasminogen activator (tPA), which are involved in hemostasis and anti-coagulation, as a potential predictor for cognitive outcomes 7 years after stroke among young stroke patients < 50 years old. The analysis of hsCRP level 3 months after stroke showed non-significant results (Pedersen et al., 2018). Just like interleukins, CRP is also a non-specific marker of inflammation with a short half-life and fast kinetics, whose level rises and falls in the setting of infections, autoimmune disorders, malignancy, necrosis, trauma, and other inflammatory conditions due to the release of pro-inflammatory cytokines such as IL-6, IL-1, and TNF-alpha (Plebani, 2023). CRP is also susceptible to ethnoracial variance, with a higher CRP level being found among Black, Hispanic, and South Asian patients when compared to White patients (Nazmi and Victora, 2007). Based on the conclusions drawn from multiple studies, CRP seems to be a weaker biomarker for late-onset PSCID when compared to results for IL-1β and IL-6 despite its association with IL-6. Further research to clarify CRP's role in PSCID and its utility as a biomarker should clarify the optimal timing of collection to improve sensitivity and specificty.

MMP-9 is an endopeptidase whose primary function is wound healing and tissue remodeling by breaking down extracellular matrix (ECM) and non-ECM targets (Pabian-Jewuła and Marcin, 2023). While MMP-9 generally serves a neuroprotective role as a remodeling enzyme (Fragkouli et al., 2014; Kaminari et al., 2017), MMP-9 overactivity during AIS contributes to disruption of the BBB, formation of vasogenic edema, and risk for hemorrhagic transformation (Chaturvedi and Kaczmarek, 2014). Furthermore, the rise of MMP-9 levels during stroke may also lead to secondary neurodegeneration through BBB disruption and secondary tissue injury, resulting in a worsened cognitive outcomes after stroke. Due to its pleiotropic neuroprotective and neurodegenerative roles, interpretation of MMP-9 levels, along with other inflammatory markers, depend largely on time of collection. Per Abdelnaseer et al., higher serum MMP-9 within 24 h after AIS was associated with worse functional outcome after 1 month post-stroke, while higher MMP-9 levels 30 days after AIS were associated with both high NIHSS at baseline and a good functional outcome at 1 month (Abdelnaseer et al., 2017). Within the group with a poor functional outcome at 1 month, mean MMP-9 was 1,111.8 ± 110.36 ng/mL within 24 h and 906.5 ± 71.43 ng/mL after 1 month; within the good functional outcome group, mean MMP-9 was 942.3 ± 143.88 ng/mL within 24 h and 747.2 ± 161.26 ng/mL after 1 month. Degree of change in MMP-9 levels from baseline to a month after stroke was neither positively nor negatively associated with patient outcome. In a study by Pu et al., elevated MMP-9 within 24 h after AIS was associated with cognitive impairment 3 months after the stroke, confirming that acutely elevated MMP-9 is associated with worse stroke outcomes (Pu et al., 2022). Mean MMP-9 in the non-cognitive impairment group was 280.6 ± 124.2 ng/mL and 392.3 ± 146.1 ng/mL in the cognitive impairment group. The authors concluded that the cut-off value of 390.7 for MMP-9 could predict the development of post-stroke cognitive impairment with 56.41% sensitivity and 81.37% specificity. The conclusions of Włodarczyk et al. were complementary to Abdelnaseer et al. in that elevated MMP-9 during the subacute phase of stroke was associated with greater cognitive recovery. When MMP-9 protein and mRNA levels were combined, they had 87% sensitivity and 71% specificity for identifying patients with cognitive improvement 4 weeks after stroke (Włodarczyk et al., 2023). Although a cut-off value was not discussed, MMP-9 levels averaged from 112.2 ± 9.7 pg/mL prior to rehabilitation and 85.4 ± 10.6 pg/mL afterwards. In whole, studies support the hypothesis that an acute rise in MMP-9 may contribute to secondary neurodegeneration, whereas elevated MMP-9 at later timepoints is associated with recovery. While the conclusions from the studies are promising, the variance in MMP-9 levels among the three discussed articles are concerning for further research prior to any clinical applications. One cause of the variance may be the small sample sizes of the discussed studies. Pu et al.'s study involved 180 patients in China, whereas Abdelnaseer et al.'s study involved 30 patients in Egypt and Włodarczyk et al.'s study included 32 patients in Poland. Hence, studies on MMP-9 and other inflammatory markers should be wary of changes in interpretation depending on the time of collection after the stroke.

## 4 Relevant biomarkers of neuroaxonal injury

Markers for neuroaxonal injury can serve as specific screening tools for PSCID arising from axonal or neuronal loss, which can be complicated by inflammatory and neurodegenerative processes. A well-known disease with pathophysiology involving neuroaxonal injury is MS, which is primarily driven by immune-mediated inflammatory degeneration of myelin and axons (Charabati et al., 2023). While several markers for neuroaxonal injury and MS have been proposed, neurofilament light chain (NfL) has emerged as the most relevant. NfL is a cytoskeletal protein forming the backbone of axons (Gaetani et al., 2019; Bacioglu et al., 2016). NfL is an intermediate filament of neurons, forming part of the cytoskeleton integral to maintaining neuronal structure (Gaetani et al., 2019). Upon axonal damage, NfL is released to the extracellular space, causing rise in CSF and plasma NfL levels that are roughly proportional to the degree of primary and secondary neuroaxonal damage (Bacioglu et al., 2016). Thus, plasma and serum NfL levels have been studied as a marker for conditions such as AD (Mattsson et al., 2017), cSVD (Egle et al., 2021; Gattringer et al., 2017), MS (Bittner et al., 2021), and primary progressive aphasia (Steinacker et al., 2017). Since infarct-associated neuro-axonal damage occurs robustly after stroke (Zhao et al., 2022), interest in the use of NfL as a biomarker for various stroke subtypes has been rising as well.

Among stroke patients, higher NfL shows a positive association with greater functional impairment after stroke (Uphaus et al., 2019) and can be a predictor of secondary neurodegeneration post-stroke (Tiedt et al., 2018). In a study by Gendron et al. (2020), 314 patients with acute cerebral infarction, ICH, and SAH were followed. High plasma NfL within the first 12–84 h post-stroke were more likely to experience functional disability by 3–6 months post-stroke. No significant finding has been found for the plasma NfL level drawn between 0 and 12 h. Other identified contributors were higher NIH Stroke Scale score and lower cognitive status at blood draw (Gendron et al., 2020). Wang Z. et al. (2021) and Uphaus et al. (2019) observed a similar pattern in their studies, where elevated plasma NfL within the first 24–48 h post-stroke was associated with cognitive impairment and functional dependence within 90 days post-stroke, respectively. Wang Z. et al. (2021) declared that the optimal threshold for prognostication was 46.12 pg/mL of plasma NfL with a sensitivity of 71.0% and a specificity of 81.5%. Uphaus et al. (2019) determined 33 pg/mL of serum NfL as a cut-off point for elevated risks of death and recurrent stroke during the median follow-up duration of 41.8 months. Pedersen et al. (2019) measured serum NfL serially after ischemic stroke to elucidate the temporal profile. The authors found that patients with high serum NfL at 3 months post-stroke were more likely to have worse neurologic and functional outcomes at 7 years. In regards to the temporal profile of NfL, Pedersen et al. concluded that the first 24 h after stroke were too early for proper analysis, while the data for the remainder of the timeline was insufficient to draw any conclusion. Although the meta-analysis by Sanchez et al. on blood NfL levels after stroke revealed a temporal pattern with a steep peak in the subacute period ~2 to 3 weeks post stroke (Sanchez et al., 2022), whether temporal patterns of NfL may provide greater prognostic value for PSCID remains to be tested. Besides its promise as a biomarker for PSCID, some studies suggest that NfL may hold diagnostic value for distinguishing stroke subtype (Sanchez et al., 2022) and prognostic value for assessing mortality, recurrent stroke risk, and cardiovascular outcomes (Uphaus et al., 2019; Gendron et al., 2020). While NfL's prognostic potential for PSCID seems promising, NfL's inter-individual and ethnoracial variability is non-negligible and would benefit from further investigation (Hviid et al., 2021; Khalil et al., 2020). In summary, elevated plasma NfL level is a promising marker for serial assessment given its temporal association with acute vascular injury and its specificity to neurons. Future research should investigate the temporal patterns that arise from different stroke subtypes to better characterize NfL's clinical sensitivity and specificity and define the optimal collection time.

## 5 Relevant biomarkers of neurodegeneration

Neurodegenerative proteins have been a popular choice for biomarker investigation due to their close relationship with the pathogenesis of neurodegenerative diseases such as AD and PD. While the exact mechanism is unclear, it has been speculated that damage due to stroke initiates or promotes a non-specific neurodegenerative process that results in pathophysiology and clinical manifestations that are similar to the progression of AD. Within the field of neurodegenerative biomarkers, three CSF and plasma markers—beta-amyloid 1-42 (Aβ42), total tau (T-tau), and hyperphosphorylated tau (P-tau) protein—have gained significant attention for their diagnostic accuracy for AD (Olsson et al., 2016; Papaliagkas et al., 2023; Verberk et al., 2018), followed by PD (Vijiaratnam and Foltynie, 2023; Liu et al., 2015) and all-cause dementia (Hosoki and Sachdev, 2024). Because of the amount and depth of the available studies on these biomarkers, as well as their relative neuronal specificity, Aβ42:Aβ40, T-tau, and P-tau may be ideal biomarkers for PSCID.

Cerebral Aβ42 and Aβ40 are amyloidogenic proteins that aggregate in the case of neurodegenerative diseases, initiating a series of events that ultimately result in AD. Because Aβ42 is more easily incorporated into aggregates than Aβ40 (Gu and Guo, 2013) and the formation of aggregates reduces the free forms of Aβ42, low CSF and plasma Aβ42:40 levels are reflective of AD (Buchhave et al., 2012; Schindler et al., 2019; Graff-Radford et al., 2007) years before the symptoms appear. Thus, if PSCID were to develop through a similar mechanism by synergism with AD pathologies, it would be logical to consider changes in the Aβ42:Aβ40 ratio prior to the onset of PSCID as well. Kang et al. (2023) followed patients who had ischemic stroke for 12 months to visually assess and found that patients who developed post-stroke cognitive impairment at 12 months had significantly higher Aβ positivity at 3 months compared to the group without cognitive impairment and the stroke-negative group. Chi et al. (2019) found that patients with post-stroke cognitive impairment a year after AIS had significantly decreased plasma Aβ42:Aβ40 at 3 months after the stroke but not at 1 week within the stroke, suggesting that Aβ42:Aβ40 measured at a later timepoint was better at predicting a 1-year outcome in the context of this study. In addition, tau protein and Aβ42 levels at 3 months post-stroke were significantly different between the two groups—one with post-stroke cognitive impairment and one without. A meta-analysis review article on Aβ1-42′s relationship with PSCID by Chen et al. (2022) draws a similar conclusion that blood Aβ1-42 levels are negatively associated with risks for cognitive impairment post-stroke. However, Huang et al. (2022) failed to note any significant differences in the plasma Aβ42:Aβ40 ratio, Aβ42 level, and Aβ40 level collected within a week after AIS among the patients who later developed cognitive impairment at year 1 compared to those who did not. Based on the currently available studies, decreased Aβ42:Aβ40 ratio measured around 3 months post-stroke seems promising for further research with goals to clarify the optimal sample measurement period. Certain drawbacks also remain to be considered. While the change in plasma Aβ42:Aβ40 level is smaller than that of CSF Aβ42:Aβ40, the drawback may be overcome by incorporating other neuronal blood-based biomarkers such as glial fibrillary acidic protein (GFAP) (Verberk et al., 2020) or APOE ε4 (Schindler et al., 2019) levels. One positive characteristic of CSF and plasma Aβ42:Aβ40 is that they possess reduced inter-individual and ethnoracial variability because they are represented as a ratio of two physiological values, improving their clinical applicability. While more research with a large sample size would be ideal, currently available evidence supports plasma Aβ42:Aβ40′s potential as a screening tool.

Tau aggregates are mainly triggered by posttranslational modifications such as phosphorylation and truncation, which causes misfolding of the tau proteins (Xiong et al., 2024). Loss of the tau protein's original structure is thought to result in the aggregates that drive neurodegenerative diseases such as AD (Hyman, 2023; Chen and Yu, 2023). Thus, CSF and plasma tau aggregate levels such as plasma T-tau are widely studied as a marker for non-specific cognitive decline, including AD and dementia (Mielke et al., 2017; Pase et al., 2019). Among P-tau subtypes, p-tau-181 (Mielke et al., 2018; Tatebe et al., 2017; Thijssen et al., 2020; Karikari et al., 2020), p-tau-217 (Barthélemy et al., 2020; Palmqvist et al., 2020; Janelidze et al., 2020), and p-tau-231 (Hampel et al., 2005) have been useful for the detection of cognitive impairment, AD, and dementia. In animal models, Mark4 up-regulation upon ischemic axonal injury can increase tau phosphorylation at Ser262 and aggregation (Hayden et al., 2019; Saito et al., 2019). Thus, it seems likely that monitoring changes in tau protein level may allow insight into tau-driven neurodegeneration after stroke. However, research on T-tau and P-tau to date is inconclusive in regards to their utility as fluid biomarkers for PSCID. In a study by Ihle-Hansen et al. (2017), a positive association was found between CSF T-tau level and the loss of brain volume a year after a stroke. The relationship reverses when measured in serum. Similar to Aβ42:Aβ40, Chi et al. (2019) concluded that there was a negative relationship between T-tau level at 3 months and development of cognitive impairment 1 year post-AIS. In another study, Huang et al. collected biomarkers within 7 days post-AIS and assessed cognitive impairment at 1 year using MoCA and Clinical Dementia Rating (Huang et al., 2022). While they found no significant difference in T-tau levels, plasma p-tau-181 at 3 months was lower in patients with cognitive impairment at 1 year compared to the patient group who maintained baseline cognitive function. Research on T-tau and P-tau may be complicated by the fact that plasma tau is susceptible to greater individual and ethnoracial variability than Aβ42 and Aβ40 (Kang et al., 2023; O'Bryant et al., 2022). Drawing conclusions from the currently available research, it seems that tau protein levels may be better suited for early-on risk assessments whereas Aβ42:Aβ40 ratio may be better fit for later assessments, and this difference in the temporal pattern of the two neurodegenerative biomarkers may also be a point of interest. However, the most impending issue is the lack of research investigating the changes in T-tau and P-tau levels post-stroke among patients who later develop PSCID. Future research on the tau proteins' potential as a biomarker for PSCID would contribute greatly to the current understanding of these proteins.

## 6 Protein interaction analysis of PSCID biomarkers

Categories of biomarkers discussed above are not mutually exclusive with each other. Inflammation is thought to influence neurodegenerative processes as suggested by disrupted pro- and anti-inflammatory balance within brains of patients with AD or PD (Kinney et al., 2018; Pajares et al., 2020). Similarly, inflammation plays a critical role in vascular injury, stroke recovery, and underlying neurodegenerative pathology that may disrupt normal recovery (Davis et al., 2003). Furthermore, elevated markers of neuroaxonal injury may be the consequence of vascular injury, inflammation-driven secondary tissue injury, or neurodegenerative mechanisms (Charabati et al., 2023). Damage to the vascular system is followed by a complex interplay of the coagulation cascade—which includes fibrinogen—and the innate immune system, which involves macrophage activation and the release of cytokines such as IL-6 and VEGF (Angelo and Kurzrock, 2007; Simon, 2012). Although biomarkers discussed within this paper were separated into four distinct, exclusive categories to facilitate discussion, the role of the markers inside a human body often involves multiple pathological mechanisms that amplify and modulate each other. Thus, we hypothesize that PSCID biomarkers of different functional categories are connected via protein-protein interactions that can be identified using an existing network analysis database.

**Figure 2:**
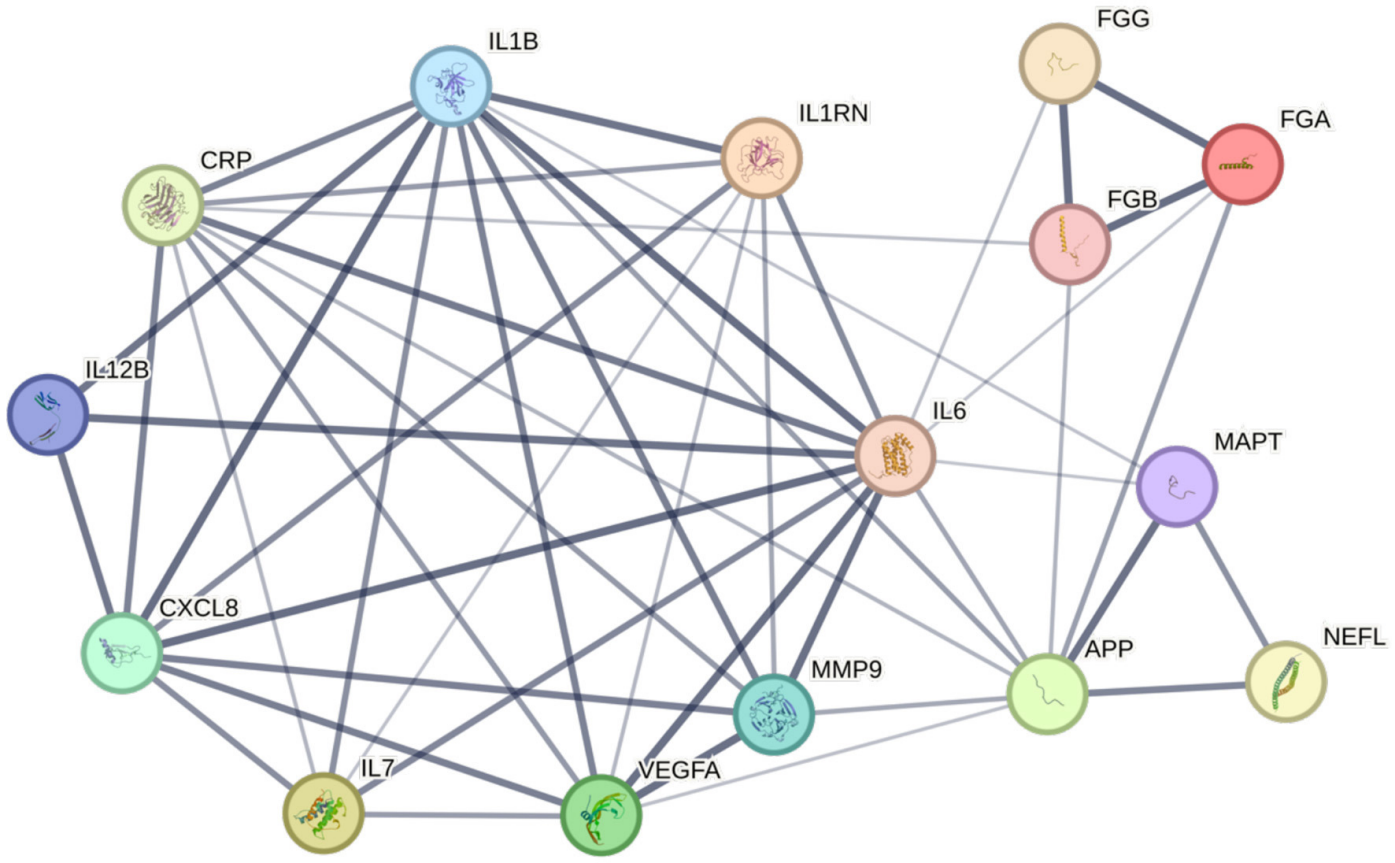
Network of fluid biomarkers. STRING database (v11.5) analysis of protein-based fluid biomarkers reveals a diagram centered on four inflammatory markers. Width of a connecting line corresponds to the confidence of interaction. *p*-value for the network is 2.99^−14^. APP, β-amyloid precursor protein; CRP, C-reactive protein; CXCL8, interleukin-8; FGA, fibrinogen alpha chain; FGB, fibrinogen beta chain; FGG, fibrinogen gamma chain; IL1B, interleukin-1 beta; IL1RN, interleukin-1 receptor antagonist protein; IL6, interleukin-6; IL7, interleukin-7; IL12B, interleukin-12 subunit beta; MAPT, microtubule-associated protein tau; MMP9, matrix metalloproteinase-9; NEFL, neurofilament light polypeptide; VEGFA, vascular endothelial growth factor A.

To identify and visualize the interconnected relationship of potential PSCID biomarkers, we used the STRING database (v11.5), whose protein database comprises of a single, representative protein per locus (Szklarczyk et al., 2019). Since fibrinogen is made of three distinct polypeptides, fibrinogen was represented as three distinct nodes—alpha chain, beta chain, gamma chain—instead of a single group. Different tau aggregates were grouped into a single microtubule-associated protein tau as STRING database does not differentiate phosphorylation status of proteins. We tested the interconnectivity of 15 proteins that reflect the putative PSCID biomarkers identified in Table 1. As a control analysis, we selected 15 additional proteins at random and tested their interconnectivity. From the group of putative PSCID blood biomarkers, we identified a significantly enriched network of 15 nodes and 48 internodal lines (*p* = 2.99^−14^) indicating a strong degree of direct protein-protein interactions among them (Figure 2). In the control STRING analysis of 15 randomly-chosen proteins, we found no significant interactions. Original and Supplementary Data for Figure 2 and the control analysis is available through permanent weblinks (Supplementary material).

The STRING database network supports the idea that pre-defined categorical classifications of PSCID biomarkers create a logical structure that is bolstered by direct protein-protein interactions and indicates that PSCID biomarkers are not independent from each other. The observed protein-protein interaction (PPI) network reveals linkage between a cluster of inflammatory biomarkers and neurodegenerative markers, particularly with β-amyloid precursor protein. Strong associations between inflammatory biomarkers and VEGF-A can also be appreciated. IL-6 had the highest number of connections at 12, followed by IL-1β which had 10 connections, and CRP and β-amyloid precursor protein which had 9 each. NfL, designated as the marker of neuroaxonal injury, has few but strong connections to neurodegenerative markers—β-amyloid precursor protein and tau protein—with high confidence, indicated by the width of the connecting lines. A major limitation of this analytic approach is that it may be biased by the imbalance in the number of studies available for each protein, meaning heavily studied proteins are more likely to have a higher number of connections.

This PPI network supports the idea of multi-factorial, complex pathophysiology leading to the development of PSCID. This also suggests that a combination of several biomarkers from two or more categories of pathogenesis may be a helpful approach in order to achieve higher diagnostic power. The approach in practice may be as simple as measuring the values of multiple biomarkers and having a set threshold for each value. However, PSCID is a multi-factorial disease process with no unique markers, and biomarkers discussed within the paper can arise from other pathologies as well, lowering their specificity to PSCID. Thus, another worthwhile approach would be to model the relative importance of each blood-based biomarker either within the protein network database or using a regression model of a large population of stroke patients to assign variable weighting and develop a PSCID risk calculator with improved accuracy.

## 7 Conclusion

While PSCID is common, the etiology, time course, and comorbid contributors are heterogeneous, which poses a challenge to identifying ideal biomarkers. Conclusive evidence for the biologic pathways driving this condition is lacking. While promising blood-based biomarkers may eventually identify patients at greatest risk for PSCID and guide management, none are currently used in routine clinical practice for this purpose. Many of the currently available studies on fluid biomarkers of PSCID are further complicated by conflicting results, small population size, low diversity, and variance in time of biomarker collection and cognitive assessment, which can heavily influence their interpretation. Despite these challenges, the present review of studies on fluid biomarkers for PSCID suggests that a combinatorial approach of two or more biomarkers of different functional categories should be tested to improve risk assessment and prognose patients with stroke. Moreover, a network-based approach with variable weighting could provide greater sensitivity and specificity for PSCID. To address this question, a large-scale study that includes harmonized biomarker collection and cognitive assessment periods is essential. The ongoing DISCOVERY study is positioned to utilize blood-based biomarkers as part of a multi-component predictive model for PSCID.
