# Supplementary material for: Linking evidence for targeted blood biomarkers in post-stroke cognitive impairment and dementia
Source: Front Stroke. 2025 Jan 7;3:1491542. doi: 10.3389/fstro.2024.1491542 (PMC12802771; doi:10.3389/fstro.2024.1491542)
Supplement: Supplementary file 1 [file Table_1.docx]

1. **Description of Supplementary Data**

In our review paper, analysis of 15 fluid biomarkers using the STRING database (v11.5) was generated to assess the level of connection and significance of protein interactions. The result shows a highly enriched network with significant protein-protein interaction enrichment (p=2.99^-14^). Full results, including exportable files generated through STRING, are accessible via the weblink below.

A control group for comparison was made by randomly selecting 15 proteins from the Homo sapiens species. The database of available human proteins (taxonomic identifier 9606) was obtained from UniProt Knowledgebase. Within the group, 15 random proteins were selected using Microsoft Excel’s random number generator function after five trials. STRING database (v11.5) analysis was performed on the chosen proteins. Full results of the analysis are accessible via the weblink below.

1. **Supplementary Data**

**Data S1. Permanent weblink to STRING network of fluid biomarkers (figure 2).**

<https://version-11-5.string-db.org/cgi/network?networkId=bbXhw7XhZAXr>

The above link leads to an interactive webpage showing the interaction network of 15 fluid biomarkers. The main figure provides a visual representation of the interactions. Nodes are able to be adjusted to better visualize the connections. Numeric and functional details of the interactions are visible within the analysis and exports tab.

**Data S2. Permanent weblink to STRING network of randomly selected proteins.**

https://version-11-5.string-db.org/cgi/network?networkId=bcGK7MAZLoR9

The above link leads to an interactive webpage showing interaction network of 15 randomly selected proteins. The main figure provides a visual representation of the interactions. No interactions are visible for this analysis.

**Data S3. Weblink to data exported to Openscience Framework.**

<https://osf.io/u46ch/?view_only=40c0e807e8a0413cb66036b28a754562>

The above link leads to an Open Science Framework (OSF) project, which contains the exportable files from the above permalinks for public access.
